# Comparison of the Inverted Internal Limiting Membrane Flap Technique and the Internal Limiting Membrane Peeling for Macular Hole with Retinal Detachment

**DOI:** 10.1371/journal.pone.0165068

**Published:** 2016-10-20

**Authors:** Takehiro Matsumura, Yoshihiro Takamura, Takeshi Tomomatsu, Shogo Arimura, Makoto Gozawa, Akira Kobori, Masaru Inatani

**Affiliations:** 1 Department of Ophthalmology, Faculty of Medical Sciences, University of Fukui, Fukui, Japan; 2 Department of Ophthalmology, Japanese Red Cross Fukui Hospital, Fukui, Japan; University of Utah (Salt Lake City), UNITED STATES

## Abstract

**Purpose:**

To evaluate the efficacy of the inverted internal limiting membrane (ILM) flap technique in vitrectomy for macular hole (MH) with retinal detachment (RD) compared with vitrectomy using ILM peeling.

**Methods:**

A retrospective case series study was performed. Twenty-two eyes of 22 patients who underwent vitrectomy for MH with RD and followed-up more than 12 months after the surgery were included in this study. We retrospectively reviewed the medical records of patients who underwent vitrectomy with inverted ILM flap technique or vitrectomy with ILM peeling. Ten patients who had been treated vitrectomy with inverted ILM flap technique, and 12 patients who had been treated vitrectomy with ILM peeling were analyzed. We evaluated changes in best-corrected visual acuity (BCVA) before and after surgery, closing rates of MH, and retinal reattachment rates and compared between both groups.

**Results:**

MH was closed and RD was reattached postoperatively in 9 eyes (90%) in the inverted ILM flap group. In the ILM peeling group, the MH was closed in 4 eyes (33.3%) and the retinas were reattached in 6 eyes (50%) after surgery. Significant improvement in BCVA after surgery (*P* = 0.0017) was only found in the inverted ILM flap group.

**Conclusions:**

Higher rates of closed MH and retinal reattachment, and small but significant improvement in BCVA were found in the inverted ILM flap group. Based on our data, the inverted ILM flap technique may be useful in vitrectomy for MH with RD.

## Introduction

Macular hole (MH) with retinal detachment (RD) can cause severe visual disorders in myopic eyes. Lower closure rates for MH with RD than in idiopathic MH can lead to poorer prognoses; therefore, additional surgical procedures may sometimes be required. Furthermore, although MH and RD were successfully closed and reattached, respectively, after vitreous surgery (providing anatomical improvement), the postoperative visual acuity may still not improve significantly [1–4].

RD resulting from MH in myopic eyes may be caused by several factors, such as the tangential macular traction attributed to premacular membranes, the presence of a posterior staphyloma, and atrophy of the retinal pigment epithelium [5]. Based on these pathogeneses, many surgical methods have been reported to improve the surgical results of treatment for MH with RD [5–10]. Vitrectomy with internal limiting membrane (ILM) removal and gas tamponade has been one of the most effective surgical procedures for RD caused by MH because that procedure can relieve tangential macular traction through complete removal of the overlying epiretinal membrane adjacent to the MH [5, 6]. In addition, the efficacy of vitrectomy with an inverted ILM flap technique has recently been reported in the treatment of idiopathic large MHs and myopic MHs [11–14]. The inverted ILM flap technique stimulates the proliferation of the glial cells that occupy MH; this enhances closure and improves the postoperative visual acuity [11, 12]. However, to the best of our knowledge, only a few cases showing the efficacy of MH closure and retinal reattachment with this procedure have been reported [12, 14].

In this study, we evaluated the results of vitrectomy with an inverted ILM flap technique for the treatment of MH with RD and compared them with the results of those after vitrectomy with ILM peeling.

## Materials and Methods

This research was a retrospective and interventional case series study which utilized data from two institutions. This study was approved by the institutional review board of University of Fukui Hospital, Fukui, Japan (approval number: 20150151). The investigations in this study adhered to the tenets of the Declaration of Helsinki. Before the surgery, a written informed consent was obtained not only for the surgery but also for use of the data for future research studies.

We retrospectively reviewed the medical records of consecutive patients who had MH with RD and who had been treated by transconjunctival 25-gauge pars plana vitrectomy with the inverted ILM flap technique between June 1, 2012 and July 31, 2015 at the University of Fukui Hospital and Japanese Red Cross Fukui Hospital. We then compared those results and the results of patients who had been treated by vitrectomy with the ILM peeling method. Most of the cases with the inverted ILM flap technique had been performed after July 26, 2013, and most of the cases with the ILM peeling had been performed between June 1, 2012 and July 25, 2013. The diagnosis of MH with RD was made by a dilated slit-lamp binocular ophthalmoscopy and spectral domain optical coherence tomography (OCT) (SPECTRALIS HRA+OCT; Heidelberg Engineering GmbH, Heidelberg, Germany).

The inclusion criteria were as follows: clinical presentation of MH with RD, 20 years of age or older, treatment with a conventional 25-gauge pars plana vitrectomy with either the inverted ILM flap technique or ILM peeling, and a follow-up period of more than 12 months after the surgery. Patients with concurrent peripheral breaks, RDs resulting from secondary MH (i.e., posttraumatic MH or due to cystoid macular edema resulting from inflammation), or proliferative vitreoretinopathy changes were excluded from this study.

All patients underwent comprehensive ophthalmologic examinations, including measurement of the best-corrected visual acuity (BCVA) and axial length. The axial length was measured preoperatively by A-scan ultrasonography (UD-6000; Tomey, Tokyo, Japan). When the axial length measurements in an eye with an RD were artifactitiously low, the error was corrected using digital calipers to evaluate the distance from the cornea to the surface of the retinal pigment epithelium rather than to the surface of the detached retina. OCT examinations were performed before and after surgery in all patients to confirm whether RD was reattached and whether MH was closed after vitrectomy. The closure of MH was defined as the absence of a neurosensory defect at the fovea [15]. The information collected retrospectively from each medical record included age, sex, axial length, preoperative lens status, presence or absence of posterior staphyloma, types of tamponade material, preoperative and postoperative BCVA, presence or absence of retina reattachment and MH closure, and intraoperative and postoperative complications.

The group that underwent a vitrectomy with an inverted ILM flap technique (inverted ILM flap group) included 10 eyes of 10 patients with MH with RD. Standard phacoemulsification and intraocular lens implantation was performed on all phakic eyes prior to vitrectomy. All eyes included in this study were treated with 25-gauge 4-port pars plana vitrectomy. All vitrectomy procedures were performed by two experienced surgeons (T.T. and A.K.).

**Fig 1:**
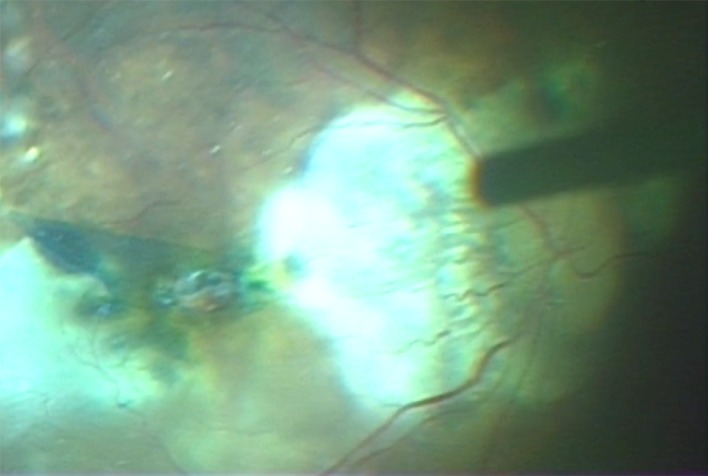
Surgical photograph of the inverted internal limiting membrane flap around the macular hole-associated retinal detachment. After brilliant blue G staining, the internal limiting membrane was peeled and inverted to cover the macular hole.

During the procedures, four cannulas were inserted obliquely and transconjunctivally into the eye using a trocar; one cannula was used for chandelier illumination. The surgical detachment of the posterior hyaloid and the excision of the central and peripheral vitreous were performed with the aid of triamcinolone (MaQaid intravitreal injection 40mg; Wakamoto Pharmaceutical Co., Ltd., Tokyo, Japan). The inverted ILM flap technique was performed according to the report by Michalewska et al [11], but with a few modifications. Briefly, the ILM was peeled off in a circular fashion for approximately 2 disc diameters around MH after a brilliant blue G (ILM-Blue; D.O.R.C. Dutch Ophthalmic Research Center B.V., Zuidland, The Netherlands) staining procedure (Fig 1). If an epiretinal membrane was present, it was peeled before the ILM peeling. During the circumferential peeling, the ILM was not completely removed from the retina but was left attached to the edge of MH. A rolled segment of the peeled ILM remained hanging in the vitreous cavity. The ILM was then massaged gently over MH from all sides until the ILM became inverted. MH was covered with the inverted ILM. After a fluid-air exchange, the air was then replaced with gas or silicone oil. The patients were advised to maintain a prone position postoperatively for at least a week. The silicone oil was used for the patients who could not keep a prone position or the severe cases including total RD, full thickness retinal folds. The silicone oil was removed 3 months after surgery after the MH closure and retinal reattachment. During the follow-up visits at 1 week and at 1, 2, 3, 6, and 12 months postoperatively, all patients underwent visual acuity measurements, slit-lamp examinations, indirect ophthalmoscopy assessments, and OCTs. The BCVAs were recorded in decimal acuity and were converted to the logarithm of the minimal angle of resolution (logMAR) units for the statistical analyses [16].

The statistical analyses were performed using JMP 10 (SAS Institute Inc., Tokyo, Japan). The statistical comparisons between the inverted ILM flap technique group and the ILM peeling group were performed using the χ2 test and the Mann–Whitney nonparametric test. Values are given as mean ± standard deviation (SD). To evaluate the surgical effects, the preoperative and postoperative BCVAs (logMAR values) were analyzed using the paired *t*-test. Values of *P* < 0.05 were considered statistically significant.

## Results

The characteristics of the 22 eyes of 22 patients who were enrolled in this study are shown in Table 1. Ten patients were treated with vitrectomy using an inverted ILM flap technique, and 12 patients were treated with vitrectomy using ILM peeling. The age, gender, mean axial length, preoperative lens status, presence of posterior staphyloma, and mean preoperative BCVA were not significantly different between both groups.

**Table 1:** Demographic data of the patients underwent vitrectomy for macular hole with retinal detachment.

	**Inverted ILM flap group (n = 10)**	**ILM peeling group (n = 12)**	***P* value**
Age (years)			
Mean (± SD)	67.7 (± 9.7)	75.3 (± 8.7)	0.11
Range	55–83	59–88	
Gender (%)			
Male	2 (20)	1 (8)	0.43
Female	8 (80)	11 (92)	
Mean axial lengths (± SD), mm	28.4 (± 2.2)	30.4 (± 1.6)	0.06
Posterior staphyloma (+ / −)	8 / 2	10 / 2	0.84
Lens status (phakic / pseudophakic)	6 / 4	5 / 7	0.51
Pre-operative BCVA, logMAR units			
Mean (± SD)	1.65 (± 0.45)	1.60 (± 0.45)	0.76
Range	0.70–2.30	1.00–2.30	
Post-operative BCVA, logMAR units			
Mean (± SD)	1.09 (± 0.35)	1.56 (± 0.36)	
Range	0.40–1.40	1.00–2.30	
Reattachment of retina (%)	9 (90)	6 (50.0)	
Closure of macular hole (%)	9 (90)	4 (33.3)	

SD, standard deviation; BCVA, best-corrected visual acuity; ILM, internal limiting membrane.

**Fig 2:**
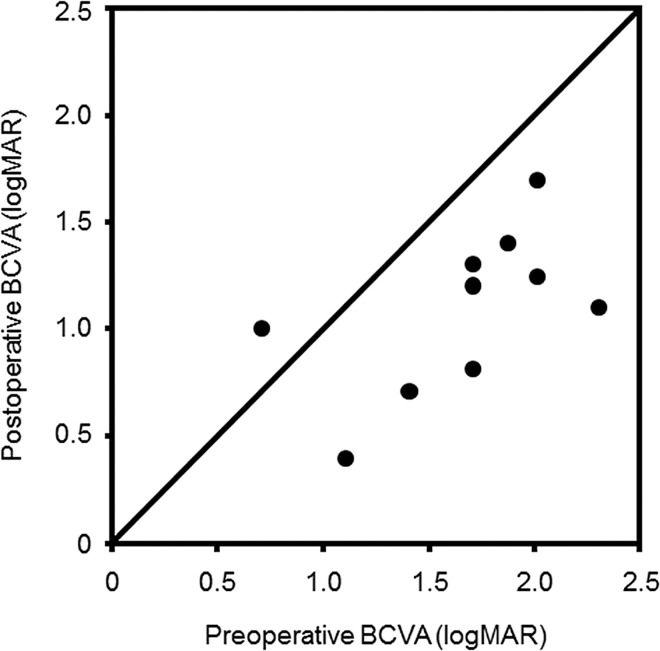
Changes in the preoperative and postoperative best-corrected visual acuities (BCVAs) of the eyes that underwent vitrectomy using the inverted internal limiting membrane flap technique for macular holes with retinal detachments. The BCVAs were converted to the logarithm of the minimal angle of resolution (logMAR) units.

In the inverted ILM flap group, the mean age of the patients (2 males and 8 females) was 67.7 ± 9.7 years (range, 55–83 years), and the mean axial length was 28.4 ± 2.2 mm. Six patients had phakic and 4 had pseudophakic eyes; phacoemulsification with intraocular lens implantation was performed in all phakic eyes prior to vitrectomy. MH closures were achieved after vitrectomy with inverted ILM flap technique in 9 of the 10 eyes (90%), and RDs were anatomically reattached in these same 9 eyes (90%) after initial surgery. The characteristics of the 10 eyes of 10 patients who were treated using the inverted ILM flap technique in this study are shown in Table 2. In the cases that used a silicone oil tamponade, the silicone oil was removed after closure of MH and reattachment of retina was confirmed after approximately 3 months. Although only one patient (No. 2) had a confirmed closure of MH and reattachment of retina, she refused the silicone oil removal operation. The procedure was unsuccessful in only one eye (No. 9); the patient then underwent a vitrectomy with autologous transplantation of the ILM [17] as the next surgery, with subsequent MH closure and RD reattachment. The mean BCVA was 1.65 ± 0.45 (range, 0.70–2.30) before surgery. In the inverted ILM flap group, the postoperative mean BCVAs were 1.40 ± 0.35 (range, 1.00–2.00), 1.26 ± 0.35 (range, 0.70–2.00), 1.21 ± 0.34 (range, 0.52–1.70), 1.20 ± 0.38 (range, 0.52–1.70), 1.16 ± 0.40 (range, 0.40–1.70), and 1.09 ± 0.35 (range, 0.40–1.40) at 1 week and at 1, 2, 3, 6, and 12 months after surgery, respectively. There was a significant difference in the mean BCVA before and 12 months after surgery (*P* = 0.0017, paired *t*-test). The changes in the preoperative and postoperative BCVAs of the eyes that underwent vitrectomy using the inverted ILM flap technique were shown in Fig 2.

**Table 2:** Characteristics of patients underwent vitrectomy with inverted ILM flap technique for macular hole with retinal detachment.

**Patients No.**	**Age**	**Sex**	**Axial length**	**Posterior staphyloma**	**Lens status**	**Tamponade**	**RD**	**MH**	**Preop BCVA**	**Postop BCVA**
1	59	F	24.36	−	Pseudophakic	SF_6_	Attached	Closed	2.30	1.10
2	58	F	31.81	+	Pseudophakic	Silicone oil	Attached	Closed	1.70	1.30
3	66	M	29.70	+	Phakic	SF_6_	Attached	Closed	1.85	1.40
4	71	F	25.77	+	Phakic	SF_6_	Attached	Closed	2.00	1.70
5	80	F	29.46	+	Pseudophakic	C_3_F_8_	Attached	Closed	1.70	0.82
6	83	F	26.52	+	Phakic	Silicone oil	Attached	Closed	1.70	1.22
7	80	F	30.25	+	Phakic	Silicone oil	Attached	Closed	2.00	1.22
8	55	M	27.19	−	Phakic	SF_6_	Attached	Closed	1.10	0.40
9	65	F	29.86	+	Phakic	SF_6_	Detached	Open	0.70	1.00
10	60	F	29.25	+	Pseudophakic	Silicone oil	Attached	Closed	1.40	0.70

ILM, internal limiting membrane; RD, retinal detachment; MH, macular hole; Preope, preoperative; Postop, postoperative; BCVA, best-corrected visual acuity.

In the ILM peeling group, the mean age of the patients (1 male and 11 females) was 75.3 ± 8.7 years (range, 59–88 years), and the mean axial length was 30.4 ± 1.6 mm. Five patients had phakic and 7 had pseudophakic eyes; phacoemulsification with intraocular lens implantation was performed on all phakic eyes prior to vitrectomy. MH closures were obtained after vitrectomy with ILM peeling in 4 of the 12 eyes (33.3%), and the retinas were reattached in 6 of the 12 eyes (50.0%) after initial surgery. The mean BCVA was 1.60 ± 0.45 (range, 1.00–2.30) before surgery. The postoperative mean BCVAs were 1.48 ± 0.33 (range, 0.82–2.00), 1.51 ± 0.29 (range, 1.10–2.00), 1.57 ± 0.28 (range, 1.10–2.00), 1.54 ± 0.27 (range, 1.10–2.00), 1.58 ± 0.33 (range, 1.00–2.00), and 1.56 ± 0.36 (range, 1.00–2.30) at 1 week and at 1, 2, 3, 6, and 12 months after surgery, respectively. Significant changes in the mean BCVA before and after surgery were not observed in the ILM peeling group.

In either the inverted ILM flap group or the ILM peeling group, the degree of changes in the preoperative and postoperative BCVAs was not significant between the eyes that underwent vitrectomy alone and the eyes that were combined with cataract surgery. The rate of silicone oil tamponade was 40.0% (4/10) and 58.3% (7/12) in the inverted ILM flap group and ILM peeling group, respectively, and no significant difference was noticed between the groups.

The closure rate of MH was significantly higher in the inverted ILM flap group than that in the ILM peeling group (*P* = 0.007, Mann–Whitney nonparametric test). The retina reattachment rate was also significantly higher in the inverted ILM flap group than in the ILM peeling group (*P* = 0.045, Mann–Whitney nonparametric test). No complications such as peripheral breaks and vitreous hemorrhage occurred during surgery or after surgery in any cases, and thus no scleral buckles were carried out.

## Discussion

The inverted ILM flap technique was initially developed to improve the functional and anatomical results in cases with large MHs; the indications for this technique were then expanded to MHs caused by high myopia [11–13]. In this study, we used the inverted ILM flap technique to treat MH with RD, and the MH closure rates after vitrectomy with this technique were significantly higher than those after vitrectomy with ILM peeling. In addition, the inverted ILM flap group had higher rates of postoperative retinal reattachment than the ILM peeling group. Although BCVA was still poor postoperatively, significant improvement in the mean BCVA was only observed after vitrectomy in the inverted ILM flap group. Our data showed that no significant differences in the improvement of visual acuity were found between the eyes underwent vitrectomy with and without cataract surgery. Thus, it is unlikely that the removal of lens opacity by the combination of cataract surgery contributes to the significant improvement of BCVA postoperatively observed in the inverted ILM flap group.

The low closure rate for MHs is one reason for intractable disease in MH with RD. Unclosed MH after surgery can cause re-detachment of the retina in the future. Based on the data in this study, the inverted ILM flap group had significantly higher MH closure rates than the ILM peeling group; therefore, the risk of retinal re-detachment and the need for additional operations would decrease after the use of the inverted ILM flap technique. In the ILM peeling group, seven cases (58.3%) required silicone oil tamponade for the prevention of RD because MHs were not closed by vitrectomy. These findings indicated that the inverted ILM flap technique had an advantage for the closure of MH with RD over the ILM peeling method. As shown in Table 2 and Fig 2, the cases with improved BCVAs after surgery were the patients who had closed MHs, and the one patient who did not have a closed MH did not have an improvement in postoperative BCVA. The closure of MH has been considered necessary for functional improvement after surgery. In fact, in the ILM peeling group, the closure rates for MH were lower than those in the inverted ILM flap group, which could be the reason that no BCVA improvement was seen after surgery in the ILM peeling group.

The peeling off of the ILM releases the macular traction and stretches the retina, which may promote closure of MH and reattachment of retinas in MH with RD. However, the MH closure rates in the ILM peeling group were lower than those in the inverted ILM flap group in this study. Because myopic eyes with RD resulting from MH often have posterior staphylomas, there may be insufficient retinal material due to the scleral elongation. The inverted ILM flap technique was effective for the treatment of idiopathic large MHs and myopic MHs through filling the hole after stimulation of glial cell proliferation. As shown in Table 1, the inverted ILM flap group had considerably higher MH closure rates, although posterior staphylomas were present in most cases (80%). The inverted ILM flap technique for MH in myopic eyes also has been reported to have high closure rates of MHs despite the presence of posterior staphylomas [12]. These findings suggested that inverted ILM tissue could support the proliferation of glial cells and could enhance MH closure in eyes with posterior staphylomas, improving the retinal reattachment rates in MH with RD.

In this study, the procedure for the inverted ILM flap technique for MH with RD was performed based on the original method for large MHs [11]. If an inverted ILM flap was peeled off of the edge of MH, autologous transplantation of the ILM would be needed similar to eyes in which the ILM had already been removed during a previous vitreous surgery. Careful procedures would be necessary for ILM trimming and removal of the epiretinal membrane because autologous ILM transplantation is more technically intricate than the inverted ILM flap technique.

An inverted ILM insertion may be an effective technique, in which the inverted ILM flap was securely trapped within MH; this technique was helpful in the closure of the MH-associated RD [18]. This modified method may reduce the risk of inverted flap dislodgement and the failure of complete MH coverage during and after surgery. However, the influence of the inserted ILM tissues, which were filled in MH from retinal surface to retinal pigment epithelium side, remains unclear over long-term periods. It may be possible to disturb the migration of glial cells and visual cells and interfere with the recovery of the retinal layer. The original surgical method may be better for the reconstruction of the retinal layer than the inverted ILM insertion method. The original method, which we performed in this study, had much higher cure rates for MH with RD than the ILM peeling method. The inverted ILM flap technique for MH with RD may be a useful vitrectomy option to improve cure rates and visual acuities during a short follow-up period.

There were several limitations to this study. First, there was a small sample size because there were not many cases of MH with RD. It was clear that further studies with a larger number of patients will be needed. Second, the improvement in BCVAs was observed over a short follow-up period, but the anatomical and functional changes in the macula remain unclear over a long-term period. Third, we were not able to evaluate the relationship between the visual acuities and the foveal microstructure in MHs that were closed after surgery. Recoveries of the ellipsoid zone and the external limiting membrane have been reported as contributors to visual acuity improvement in cases of MHs after surgery [19, 20]; however, we could not precisely confirm the restoration of the retinal layer lines in patients with MH-associated RD. These myopic patients had very thin retinas and choroids, and it was difficult to investigate these lines even with spectral domain OCT.

In conclusion, higher MH closing rates and retinal reattachment rates and small but significant postoperative improvement of visual acuity were found in the group using the inverted ILM flap technique over a short follow-up period. The inverted ILM flap technique for MH with RD may be a more useful vitrectomy option than the ILM peeling method.
